# Environmental reservoirs of the drug-resistant pathogenic yeast *Candida auris*

**DOI:** 10.1371/journal.ppat.1011268

**Published:** 2023-04-13

**Authors:** Ayorinde B. Akinbobola, Ryan Kean, Syed Manzoor Ahmed Hanifi, Richard S. Quilliam

**Affiliations:** 1 Biological and Environmental Sciences, Faculty of Natural Sciences, University of Stirling, Stirling, United Kingdom; 2 Department of Biological and Biomedical Sciences, School of Health and Life Sciences, Glasgow Caledonian University, Glasgow, United Kingdom; 3 International Centre for Diarrhoeal Disease Research, Bangladesh (icddr,b), Health System and Population Studies Division, Shaheed Tajuddin Ahmed Sarani, Mohakhali, Dhaka, Bangladesh; Carnegie Mellon University, UNITED STATES

## Abstract

*Candia auris* is an emerging human pathogenic yeast; yet, despite phenotypic attributes and genomic evidence suggesting that it probably emerged from a natural reservoir, we know nothing about the environmental phase of its life cycle and the transmission pathways associated with it. The thermotolerant characteristics of *C*. *auris* have been hypothesised to be an environmental adaptation to increasing temperatures due to global warming (which may have facilitated its ability to tolerate the mammalian thermal barrier that is considered a protective strategy for humans against colonisation by environmental fungi with pathogenic potential). Thus, *C*. *auris* may be the first human pathogenic fungus to have emerged as a result of climate change. In addition, the release of antifungal chemicals, such as azoles, into the environment (from both pharmaceutical and agricultural sources) is likely to be responsible for the environmental enrichment of resistant strains of *C*. *auris*; however, the survival and dissemination of *C*. *auris* in the natural environment is poorly understood. In this paper, we critically review the possible pathways through which *C*. *auris* can be introduced into the environment and evaluate the environmental characteristics that can influence its persistence and transmission in natural environments. Identifying potential environmental niches and reservoirs of *C*. *auris* and understanding its emergence against a backdrop of climate change and environmental pollution will be crucial for the development of effective epidemiological and environmental management responses.

## Author summary

The natural environment can play a significant role in the epidemiology of infectious diseases as pathogens persist and evolve in environmental niches from where they can be transferred to new hosts. Therefore, understanding the interaction of pathogens with potential environmental reservoirs is crucial to the control of pathogenic diseases. Pathogenic *Candida* species have been widely isolated from nonclinical environmental sources such as soil, freshwater, seawater, and animals such as birds. The persistence and dissemination of *C*. *auris*, a recently emerged human pathogenic yeast, in the environment is still poorly understood. Here, we identify the potential pathways through which *C*. *auris* can be introduced into the natural environment and the characteristics that can influence its survival in various environmental matrices. In conclusion, we review the current knowledge gaps and identify the future research needed to understand the epidemiological implications of *C*. *auris* in the natural environment.

## Introduction

**Fig 1:**
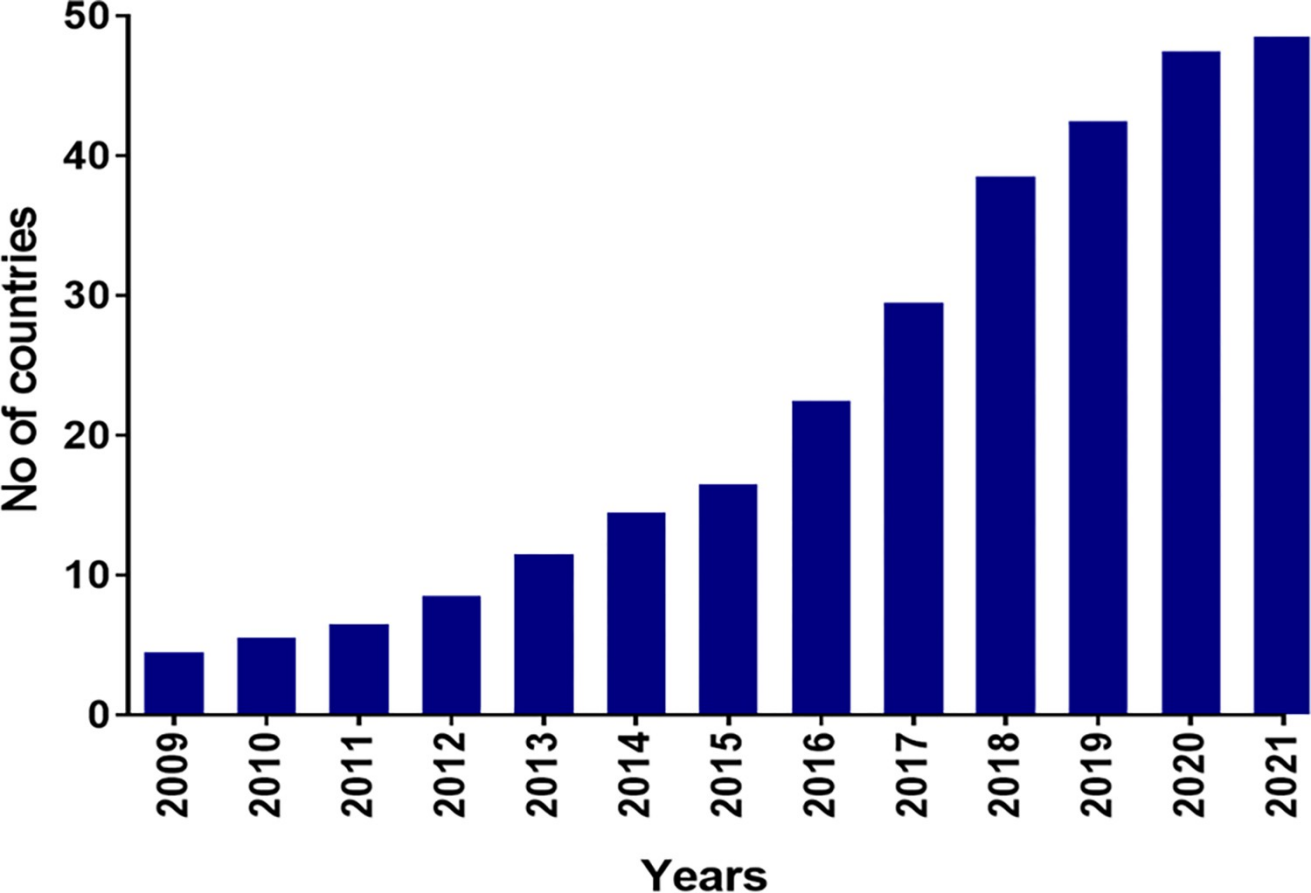
Cumulative number of countries with reported detection of *Candida auris*.

*Candia auris* is a human pathogenic yeast commonly resistant to multiple antifungal drugs. The first report of *C*. *auris* being isolated was in 2009 from the ear of a 70-year-old patient in Tokyo, Japan [1]; however, retroactive detection of *C*. *auris* has subsequently been reported from samples collected in South Korea, Japan, and Pakistan [2–4]. Since 2014, there has been a sharp rise in the number of countries reporting *C*. *auris* detection for the first time (Fig 1) and it has now been detected in more than 45 countries [5–8]. This global spread of *C*. *auris* has prompted the Centres for Disease Control and Prevention (CDC) to discontinue updating data on first case detection in new countries [8]. Increasing global temperatures have been implicated for the almost simultaneous emergence of this pathogen on 3 continents [9]. Thus, it has been hypothesised that *C*. *auris* is the first human pathogenic fungus to have emerged as a result of climate change [9,10].

Pathogenic *Candida* species cause candidiasis, which can range from superficial conditions such as oral thrush to life-threatening invasive diseases. Globally, candidiasis is the third most common healthcare-associated infection with invasive candidiasis responsible for about 20% of infections in intensive care units worldwide [11,12]. While there are millions of new cases of candidiasis of the mucosa each year, the annual global incidence of life-threatening invasive candidiasis is approximately 750,000 and is associated with a high mortality rate, particularly in critically ill patients and those with compromised immunity [11,13]. Although *Candida albicans* is the most common aetiological agent of candidiasis, there has been an increase in cases of candidiasis caused by a range of other *Candida* species such as *C*. *glabrata*, *C*. *parapsilosis*, and more recently *C*. *auris* [11,12,14].

*C*. *auris* infections, like many other *Candida* infections, are opportunistic [15]. Underlining health conditions such as diabetes, kidney disease, and HIV/AIDS can be risk factors for *C*. *auris* infections [5,16,17], as can long-term use of antimicrobial drugs, extended hospital stays, surgery, and the use of a central venous catheter [5,16]. Recently, Coronavirus Disease 2019 (COVID-19) has also been suggested as a risk factor for severe *C*. *auris* infection based on the high mortality rate recorded in COVID-19 patients with *C*. *auris* infection [6,18]. Although a meta-analysis of available data on COVID-19–associated *C*. *auris* infections indicated that COVID-19 has no significant impact on the prevalence of *C*. *auris* infection [19,20].

An emerging resistance to multiple antifungal drugs (such as fluconazole and amphotericin B) and the ability to persist in nosocomial settings (together with poor prognoses) have made *C*. *auris* infections a serious global health concern. The CDC and the European Centre for Disease Control and Prevention (ECDC) have both declared *C*. *auris* an urgent health threat that needs a prompt and vigorous response [21,22]. Likewise, the World Health Organisation (WHO) have recently classified *C*. *auris* as a fungal pathogen of critical concern on its “fungal pathogen priority list” compiled to guide research, development, and public health action [23]. Infection control measures stipulate colonisation screening if a case of *C*. *auris* is detected in healthcare facilities, and many national guidelines now require cases of *C*. *auris* infection to be reported to central infection control agencies [21,22,24]. Although *C*. *auris* has largely been isolated in healthcare facilities, there have also been limited cases of asymptomatic community colonisation [5]. However, the emergence of *C*. *auris* is likely to be a fairly recent event as it has not been retroactively detected in collections of fungi isolated from humans prior to 1996 [25].

In contrast to pathogenic moulds and non-*Candida* human pathogenic yeast (such as *Cryptococcus neoformans*), which are known to inhabit diverse environmental niches, pathogenic *Candida* species primarily exist as human commensals or as contaminants in clinical environments from where they can cause opportunistic infections. However, several species of pathogenic *Candida* have been isolated from nonhuman or nonclinical environmental samples [26,27], which could provide novel exposure routes for human infection [26,28–30]. *Candida albicans* was previously thought to exist mainly as a human commensal [30], but has now been widely isolated from nonclinical environmental samples, e.g., soil, wetlands, and plants (Table 1). In addition, the 5 most common pathogenic *Candida* species [31] as well as *C*. *lusitaniae* and *C*. *haemulonii*, 2 human pathogenic *Candida* species closely related to *C*. *auris* [5], have also been isolated from nonclinical environmental samples (Table 1). Despite the common isolation of pathogenic *Candida* species from environmental samples, the significance of environmental persistence and cycling on the epidemiology of candidiasis is not yet fully understood. Therefore, identifying potential environmental niches and reservoirs of *C*. *auris* is crucial for the development of effective epidemiological and management responses.

**Table 1:** Reported isolation of common pathogenic *Candida* species from environmental samples.

*Candida* species	Environmental sample	References
***Candida albicans***	Soil, freshwater, seawater, plants, fruit, decaying organic matter, bird droppings	[29,30,32–37]
***Candida parapsilosis***	Soil, woody debris, fruits, bird droppings, freshwater, seawater, estuary, marine invertebrates	[29,32,34–44]
***Candida tropicalis***	Soil, fruits, wood, freshwater, seawater, marine invertebrates	[28,29,34,36–39,41–45]
***Candida glabrata***	Soil, fruit, freshwater, seawater, estuary	[29,32,34,37,39,40]
***Candida krusei***	Fruits, freshwater	[34,38,39,42]
***Candida lusitaniae***	Bird droppings, plants, insects	[46–50]
***Candida haemulonii***	Arthropods, plants, soft corals, seawater	[51–54]

Recent studies have increased our understanding of the biology, genome, pathogenicity, and phenotypic characteristics of *C*. *auris* [5,16,55], yet the role of the natural environment in the emergence and transmission of *C*. *auris* remains largely unexplored. The environment can provide suitable conditions for human pathogens to persist and provides a diversity of human exposure routes (either directly or via secondary hosts or fomites). Importantly, environmental interactions can also lead to the acquisition of both novel genes and phenotypic characteristics that can significantly impact pathogenicity [56,57]. Therefore, the aim of this paper is to determine potential interactions of *C*. *auris* with the environment and explore those environmental characteristics and pathways that could facilitate increased human exposure to *C*. *auris*.

## Sources and input pathways of *Candida auris* in the environment

**Fig 2:**
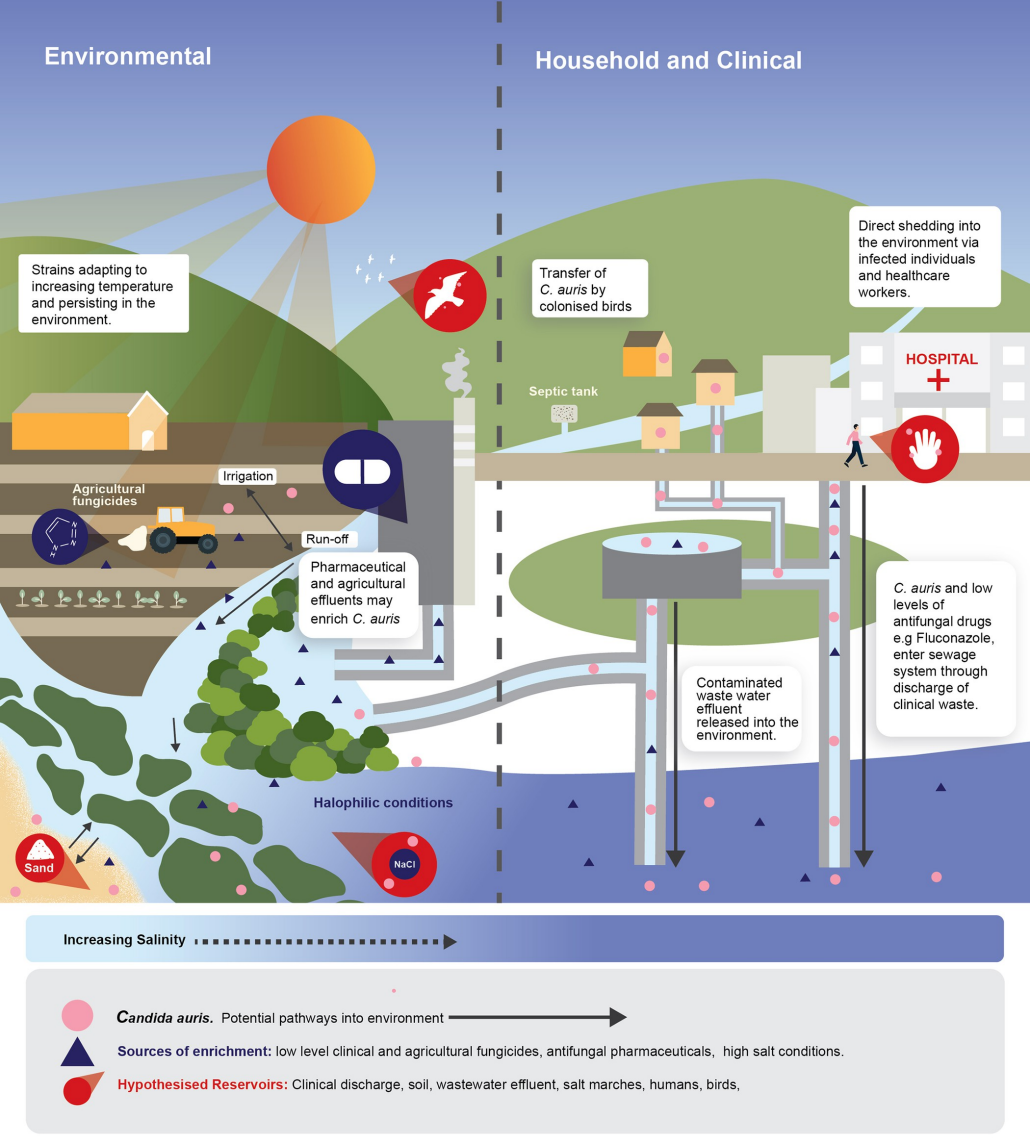
Possible pathways for *Candida auris* introduction into the environment.

Clinical strains of *C*. *auris* can be introduced into the environment through shedding from colonised humans or animals or through the release of contaminated clinical wastes (Fig 2). *C*. *auris* persists on the skin, nostrils, and ear cavity of colonised humans, which are widely documented routes for contamination of clinical environments by infected patients and healthcare workers [58,59]. The detection of *C*. *auris* in indoor swimming pools demonstrates that colonised individuals can shed the yeast into nonclinical environments, where the cells can persist and be subsequently transferred to other people [60]. *C*. *auris* has been detected in human urine and faecal samples [4,61], as well as rectal swabs from both symptomatic and asymptomatic individuals [62]. Thus, open defecation, which is widely practiced in rural communities in low- and medium-income countries, could also be a significant pathway for the environmental loading of *C*. *auris*. Likewise, *C*. *auris* in human excreta could contaminate the environment via effluent from wastewater treatment plants. Although *C*. *auris* detection in wastewater has not yet been reported, other pathogenic yeasts such as *C*. *albicans* are often detected in hospital wastewater [63] and in the influent and effluent of wastewater treatment plants [64]. In all cases, the environmental survival and subsequent persistence of *C*. *auris* will be affected by the intrinsic characteristics of the receiving environment following wastewater discharge.

Contaminated clinical solid waste is a proven pathway for the introduction of pathogenic microorganisms into the environment [65], and clinical wastes contaminated with pathogenic species of *Candida*, e.g., *C*. *albicans*, *C*. *glabrata*, and *C*. *parapsilosis*, have previously been reported [66]. *C*. *auris* shows an increased ability to persist on dry inanimate surfaces compared to *C*. *albicans* [67] and can persist in biofilms on inanimate surfaces, e.g., plastics, for significant periods [68–70]. In developing countries where clinical wastes are often not properly managed due to limited resources, safe disposal of contaminated wastes remains a serious challenge.

Although no animal reservoir has yet been established for *C*. *auris*, this thermotolerant yeast can colonise warm-blooded nonhuman animals, for example, human pathogenic *Candida* species are often isolated from birds, with subsequent faecal shedding into the environment [71,72]. The most common pathogenic *Candida* species (e.g., *C*. *albicans*, *C*. *glabrata*, *C*. *tropicalis*, *C*. *parapsilosis*, and *C*. *krusei*) have been isolated from bird droppings [73], and other pathogenic yeasts have been isolated from mammals such as bats and dogs [74,75].

## *Candida auris* persistence and cycling in the environment

Populations of *C*. *auris* have both thermotolerant and halotolerant capacity and are commonly resistant to one or more antifungals [68,76,77]. Based on these phenotypic attributes, it has been suggested that *C*. *auris* probably emerged from a natural reservoir [25,70,78,79], which is supported by genomic evidence, and the ecology of related fungal species; however, specific reservoirs of *C*. *auris* in the natural environment have still not been properly defined. Potential environmental reservoirs for *C*. *auris* include terrestrial, freshwater, and marine ecosystems (Table 2), with specific niches in soil, plants, and animals. *C*. *auris* can tolerate high salt concentrations (of up to 10% NaCl) [77], and evidence suggests that human pathogenic *C*. *auris* evolved in niches in marine ecosystems [25,78,80]. Support for this comes from isolates of *C*. *auris* that have recently been discovered in an area of coastal wetland in the Andaman Islands with no known human activity and from an estuary in Colombia [80,81].

**Table 2:** Potential reservoirs of *Candida auris*.

Source	Suggested reservoir	Hypothesis/evidence	Reference
**Environment**	Soil	Acquisition of virulence attributes through survival in harsh soil conditions Adaptation to fungicide in soil Adaptation to higher temperature in soil niches due to global warming Reported isolation of closely related species	[25,70,78]
	Marine ecosystem	Halotolerant ability of *C*. *auris* Reported isolation of closely related species Isolation from water collected from sandy beach	[78,80]
	Coastal wetlands	Adaptation to higher temperature due to global warming Isolation from salt marsh sediment and wetland sandy beach	[25,78,80]
	Estuary	Detection in water samples collected from estuary	[81]
**Animals**	Birds	Thermotolerant ability of *C*. *auris* Extensive isolation of other pathogenic *Candida* species from birds	[25,78]
	Aquatic animals	Reported isolation of closely related species	[78]
	Insects	Reported isolation of closely related species	[78]
**Plants**	Plants	Reported isolation of closely related species	[25,78]
	Fruits	Detection on stored fruits	[79]
**Human**	Human population	Ability to successfully colonise human skin Reported isolation of closely related species	[78]

*C*. *auris* has the ability to grow at temperatures of up to 42°C, where phylogenetically related species, e.g., *C*. *haemulonii*, cannot grow [82]. The thermotolerant characteristics of *C*. *auris* have been attributed to environmental adaptation to increasing temperature due to global warming. It is hypothesised that this has facilitated its emergence as a human pathogen due to its ability to tolerate the mammalian thermal barrier, which is considered a protective strategy for humans against colonisation by environmental pathogens [9,83]. The ability of *C*. *auris* to tolerate higher temperatures may also enable the colonisation of birds, which has been suggested as a transmission pathway from environmental reservoirs to humans [25,84]. Although there is currently no direct evidence of *C*. *auris* isolation from birds, the detection of other human pathogenic *Candida* species in birds has been widely reported [85,86].

Phenotypic characteristics that support the hypothesis of *C*. *auris* emergence from an environmental niche include the intrinsic resistance to antifungal drugs (most commonly fluconazole) by most *C*. *auris* isolates. The large-scale introduction of antifungal chemicals such as azoles into the environment (from both pharmaceutical and agricultural sources) has likely played a role in the environmental enrichment of fluconazole resistant strains of *C*. *auris* [70,78]. Azole antifungal agents are widely used to control fungal crop diseases and thus inadvertently contaminate the environment and can persist for months in different environmental matrices [87,88]. Similarly, it has been hypothesised that increasing the shelf life of stored fruits with antifungal chemicals has led to the selection of antifungal resistance in environmental *C*. *auris* strains [79]. Comparable selection pressure from antifungals in the environment has been associated with the emergence of azole-resistant *Aspergillus fumigatus* [89].

Genomic evidence also supports the hypothesis that *C*. *auris* originated from, and diversified in, environmental reservoirs. For example, there is a high occurrence (i.e., tens of thousands) of single nucleotide polymorphisms (SNPs) in the 5 *C*. *auris* clades that have originated in different geographical locations in South America, South Africa, South Asia, East Asia, and most recently in Iran [16,55,90], in contrast to the low (i.e., less than 70 SNPs) intra-clade genetic diversity [16]. The high inter-clade genomic difference has been attributed to the selective pressure of specific environmental conditions at the different geographical locations where each *C*. *auris* clade emerged. The *C*. *auris* genome also provides evidence for the environmental selection of antifungal resistance genes in *C*. *auris* [91]. Recently, searches of the Sequence Read Archive of the NCBI database detected *C*. *auris* sequences from historic environmental samples [92] and identified 7 *C*. *auris* metabarcoding datasets, some of which were obtained from environmental samples collected from different parts of the world. Taken together, the available evidence strongly supports the hypothesis that *C*. *auris* emerged from reservoirs in the natural environment under various environmental stress including selection pressure from antifungals.

Soil can support the survival of a wide range of human pathogens, including *C*. *albicans* that can persist in soil for more than 30 days [30]; however, the ability of *C*. *auris* to persist in soil has not yet been examined. Lower pH and higher minerals (aluminium, manganese, and sodium) can increase survival of *C*. *albicans* during the early stages of soil contamination, while cation exchange capacity (CEC) and clay content, which influences the exchangeable minerals in soil, appears to be crucial for longer-term survival in soil [30]. *C*. *auris* can thrive between a pH range of 4 to 13, [93] and can withstand cationic stress, so could potentially persist in high mineral soils. The ability of *C*. *auris* to persist in biofilms in water-limited conditions [67,68] indicates that it could survive desiccation in soil; however, the persistence of *C*. *auris* in soil under a range of environmental conditions is still unknown.

Pathogenic species of *Candida* have been widely isolated from freshwater sources [39,94,95], although the only reported isolation of *C*. *auris* is from the water of an indoor swimming pool in the Netherlands [60]. *C*. *albicans* can persist for significant periods in filtered or sterile water [96,97], although these studies do not account for the influence of background microbial communities and other biotic factors that would be present in environmental freshwaters. Microbial diversity in freshwater is influenced by biotic factors and a wide range of abiotic components, e.g., nutrient availability, dissolved oxygen, pH, temperature, all of which are subject to wide variations [98]. Yeasts are generally tolerant of environmental stressors, although it has been demonstrated that *C*. *auris* has a poor ability to survive in anaerobic conditions compared to other pathogenic *Candida* species like *C*. *albicans* and *C*. *glabrata* [99], which may affect its ability to persist in freshwater environments with low dissolved oxygen.

A major determinant of the ability of microbial species to persist in marine environments is the ability to withstand cationic stress due to high salt concentration, and several species of *Candida* species are frequently found in marine environments [37,41,44,45,100,101]. The halotolerance of *C*. *auris* (and its hypothesised emergence from the marine environment) could facilitate the survival of *C*. *auris* and its subsequent dissemination over large distances [77,80,99].

## Future research focus

Current knowledge of *C*. *auris* persistence has focused on clinical settings due to the potential for infection outbreaks in healthcare settings. However, knowledge of the survival and dissemination of *C*. *auris* through the environment is now urgently needed to give a more complete assessment of the risk of *C*. *auris* transmission from environmental reservoirs to humans. The focus of future research on *C*. *auris* in the environment should be divided into 2 broad themes: (1) understanding environmental distribution and dissemination; and (2) quantifying survival and persistence in the environment.

There is a need to develop environmental surveillance tools to rapidly detect and quantify *C*. *auris*, but importantly, these must be developed in tandem with tools for community-scale surveillance. Tools such as wastewater-based epidemiology, which has been so effective for surveillance of diseases like COVID-19 [102] allows an understanding of community levels of infection (including asymptomatic carriage). *C*. *auris* contaminated wastewater from healthcare facilities may be discharged into the environment if *C*. *auris* is able to survive wastewater treatment processes. Although there are no reports of *C*. *auris* detection in wastewater, it is often detected in clinical faecal samples and other pathogenic *Candida* species, including *C*. *albicans*, *C*. *glabrata*, *C*. *krusei*, *C*. *tropicalis*, and *C*. *dubliniensis*, have been detected at different wastewater treatment stages [103]. However, effective surveillance is not possible without specific detection methods. Most of the currently available methods have primarily focused on the detection of *C*. *auris* in clinical samples rather than complex environmental samples [104]. Therefore, before surveillance methods can be widely applied, there is an urgent need to optimise the specificity of both culture-dependent and culture-independent methods to detect *C*. *auris* in samples that may contain relatively high levels of other *Candida* species. This is especially important as the difficulty of distinguishing *C*. *auris* from other closely related *Candida* species has previously led to the misidentification of *C*. *auris* [2,16].

While *C*. *auris* can tolerate stresses associated with the infection process (physiological stress such as oxidative stress and cationic stress) and from colonising clinical surfaces (disinfection, temperature, and cell wall stress), the ability of *C*. *auris* to tolerate environmental stresses such as solar radiation, salinity, temperature extremes, competition, and predation have not yet been examined under natural environmental conditions. The ability of *C*. *auris* to tolerate environmental stressors will determine its fate when released into the environment and its potential for subsequent dissemination pathways and human exposure. A common strategy used by microorganisms to resist environment stress is to form biofilms. *C*. *auris* are known for their ability to form persistent biofilms [69,105] and as such could form multispecies biofilms with other environmental fungi and bacteria species, e.g., *Staphylococcus* spp. [106]. It has recently been demonstrated that free-living protozoans commonly found in hospital water pipes can enhance the persistence of *C*. *auris* [107]. Clearly, the impact of such close interactions with microbial species in multispecies biofilms on the persistence and pathogenicity of *C*. *auris* in the environment is an area that needs urgent investigation.

Although the environmental reservoirs for *C*. *auris* are not yet well known, the release of antifungals into the environment from activities such as agricultural usage and industrial waste disposal could be facilitating novel reservoirs in the environment. Importantly, drug resistance could theoretically be transferred between compatible mating types of *C*. *auris*, or even with other species of *Candida*, leading to the potential for novel antifungal and pathogenicity genotypes with increased virulence. A recently recognised phenomenon for the transport of human pathogens in the environment (and the exchange of antimicrobial resistance genes) is their ability to attach and persist on plastic wastes in terrestrial and aquatic ecosystems [108,109]. Plastic wastes are increasingly being introduced into the environment and can be dispersed over long distances. *C*. *auris* can colonise and persist on plastic surfaces in healthcare settings for more than 28 days [68]; therefore, the ability of *C*. *auris* to persist on environmental plastic pollutants (e.g., from clinical waste) and be disseminated within the environment could be a novel transport mechanism for spreading the pathogen.

The natural environment is an important component of the infection cycle for a wide variety of pathogenic diseases, and *C*. *auris* has various potential pathways of introduction into the environment. Understanding the environmental interactions of *C*. *auris* will provide the insight required to understand the role that the natural environment played in the emergence of these pathogenic fungi and provide a better understanding of the factors that can influence the persistence and dissemination of *C*. *auris* in the environment.
